# Surface Deformation of Xiamen, China Measured by Time-Series InSAR

**DOI:** 10.3390/s24165329

**Published:** 2024-08-17

**Authors:** Yuanrong He, Zhiheng Qian, Bingning Chen, Weijie Yang, Panlin Hao

**Affiliations:** 1 Big Data Institute of Digital Natural Disaster Monitoring in Fujian, Xiamen University of Technology, Xiamen 361024, China; 2012112001@xmut.edu.cn (Y.H.); 13394716601@163.com (B.C.); 15931185171@163.com (W.Y.); hpl0621@gmail.com (P.H.); 2 Hunan Key Laboratory of Remote Sensing Monitoring of Ecological Environment in Dongting Lake Area, Changsha 410004, China

**Keywords:** land subsidence, Xiamen, PS-InSAR, SBAS-InSAR, cause analysis

## Abstract

Due to its unique geographical location and rapid urbanization, Xiamen is particularly susceptible to geological disasters. This study employs 80 Sentinel-1A SAR images covering Xiamen spanning from May 2017 to December 2023 for comprehensive dynamic monitoring of the land subsidence. PS-InSAR and SBAS-InSAR techniques were utilized to derive the surface deformation field and time series separately, followed by a comparative analysis of their results. SBAS-InSAR was finally chosen in this study for its higher coherence. Based on its results, we conducted cause analysis and obtained the following findings. (1) The most substantial subsidence occurred in Maluan Bay and Dadeng Island, where the maximum subsidence rate was 24 mm/yr and the maximum cumulative subsidence reached 250 mm over the course of the study. Additionally, regions exhibiting subsidence rates ranging from 10 to 30 mm/yr included Yuanhai Terminal, Maluan Bay, Xitang, Guanxun, Jiuxi entrance, Yangtang, the southeastern part of Dadeng Island, and Yundang Lake. (2) Geological structure, groundwater extraction, reclamation and engineering construction all have impacts on land subsidence. The land subsidence of fault belts and seismic focus areas was significant, and the area above the clay layer settled significantly. Both direct and indirect analysis can prove that as the amount of groundwater extraction increases, the amount of land subsidence increases. Significant subsidence is prone to occur after the initial land reclamation, during the consolidation period of the old fill materials, and after land compaction. The construction changes the soil structure, and the appearance of new buildings increases the risk of subsidence.

## 1. Introduction

Surface deformation refers to changes in the shape and position of the Earth’s surface. Land subsidence, a kind of surface deformation, is the gradual reduction in ground elevation. This process, triggered by natural or human-induced factors, can pose destructive hazards [1,2,3]. Over the past few decades, a large number of countries around the world, such as China, Mexico, and Indonesia, have all suffered significant land subsidence, resulting in tremendous economic losses [4,5,6]. In China, incomplete statistics indicate that, until 2021, land subsidence of varying degrees has been observed in 113 cities at and above the prefectural level. The subsidence occurring in cities like Shanghai, Beijing, Tianjin, and Xi’an is extremely serious [7,8,9]. During rapid urbanization, land subsidence emerges as a predominant geological hazard faced by cities [10]. It is imperative to monitor urban surface deformation to mitigate losses resulting from such geological disasters.

Conventional methods for monitoring surface deformation are leveling [11], ground photogrammetry [12], laser scanning [13], and Global Navigation Satellite System (GNSS) [14,15,16] applications. However, these methods suffer from many limitations, including high costs, extended monitoring durations, vulnerability to adverse weather conditions, risks associated with manual fieldwork, and an inadequate comprehension of the ground surface characteristics [17]. Interferometric synthetic aperture radar (InSAR) technology combines synthetic aperture radar with interferometric methods [18]. Compared with traditional surface deformation monitoring methods, it offers several advantages, including low cost, short monitoring periods, high accuracy, extensive coverage, and all-weather operation capability [19]. Consequently, it has become an increasingly popular technique for monitoring urban surface deformation [20].

Several InSAR techniques have been employed in urban surface deformation monitoring research, such as differential interferometric synthetic aperture radar (D-InSAR) [21], persistent scatterer InSAR (PS-InSAR) [22,23], and small baseline subsets InSAR (SBAS-InSAR) [24], with PS-InSAR and SBAS-InSAR being the most commonly utilized [25,26,27]. Hu [28] et al. used both SBAS-InSAR and PS-InSAR techniques to monitor the surface deformation in Nanchang City, China. The results are highly correlated, indicating significant surface deformation variations in parts of Nanchang being influenced by precipitation changes. Hussain [26] et al. employed the PS-InSAR technique to monitor land subsidence in the groundwater extraction zones of Lahore, Pakistan, finding high susceptibility in regions with substantial groundwater extraction and sedimentary soil, exacerbated by construction activities. Zhao [29] et al. utilized the SBAS-InSAR technique to monitor land subsidence in Wuhan, China, between 2017 and 2021, linking the subsidence center to subway construction and building development, particularly in regions with carbonate formations and soft soil covers susceptible to karst collapse and land subsidence. Ao [4] et al. conducted a systematic assessment of land subsidence in major Chinese cities from 2015 to 2022 using the InSAR technique, and found that subsidence appears to be related to a range of factors, such as groundwater abstraction and building weight.

Xiamen, located in a seismic zone along China’s southeastern coast, is prone to geo-logical hazards. Despite rapid urbanization, the construction of numerous buildings and infrastructure, and its large population, comprehensive surface deformation monitoring in Xiamen has been limited. For example, Zheng [30] et al. analyzed the land subsidence and spatial differences in Xiamen from 2001 to 2015 by using elevation information, and also discussed the impact of construction land, building plot ratio and land use conversion information on the land subsidence in Xiamen. The elevation information has limitations in capturing subtle subsidence changes, and they discussed relatively few influencing factors. The existing research on the driving factors of land subsidence in Xiamen mostly focuses on the reclamation of the new airport in Xiang’an District. For example, Zhuo [31] et al. used the InSAR technique to analyze the subsidence evolution of the new airport during the reclamation period. The analysis showed that the airport has severely subsided due to the compaction of soil and sand. Li [32] et al. studied the deformation history of the new airport through the DS-InSAR method, revealing the deformation caused by land reclamation. Xiong [33] et al. used the MT-InSAR method to process the Sentinel-1 images covering the new airport. The results showed that the subsidence mainly occurs in the reclamation area, and the maximum average subsidence rate exceeds 30 mm/y. They also predicted the consolidation time, finding that the deformation rate is positively correlated with the consolidation time. There are relatively few studies on the driving factors such as subway lines. For example, Tan [34] et al. set up a large number of monitoring points to monitor the deep foundation pit of Maluan North Station of Xiamen Metro Line 2, and through numerical simulation, it was found that the land subsidence at the corner of the pit shows obvious corner effects. However, research on other driving factors of land subsidence in Xiamen, such as geological structure, groundwater exploitation, and precipitation, has not been found. Currently, people have a limited understanding of the subsidence trend and rate of the entire Xiamen and the driving factors of subsidence. Further analysis is needed to understand the deformation characteristics caused by natural and human factors. This study is the first to comprehensively and systematically explore the influencing factors of surface subsidence in Xiamen with the InSAR technique.

This study addresses the research gap by employing PS-InSAR and SBAS-InSAR techniques to extensively monitor the land subsidence in the whole city, using 80 Sentinel-1A satellite images spanning from May 2017 to December 2023. The deformation field and time series obtained by these techniques were compared and analyzed. Then, we extracted typical subsidence zones from the optimal surface deformation field. On this basis, we investigated the spatiotemporal driving factors of land subsidence using the long-term time series. This research supports disaster mitigation, urban planning, engineering safety, and environmental conservation in Xiamen, providing a reference for urban development in other cities.

## 2. Study Area and Data

### 2.1. Study Area

**Figure 1:**
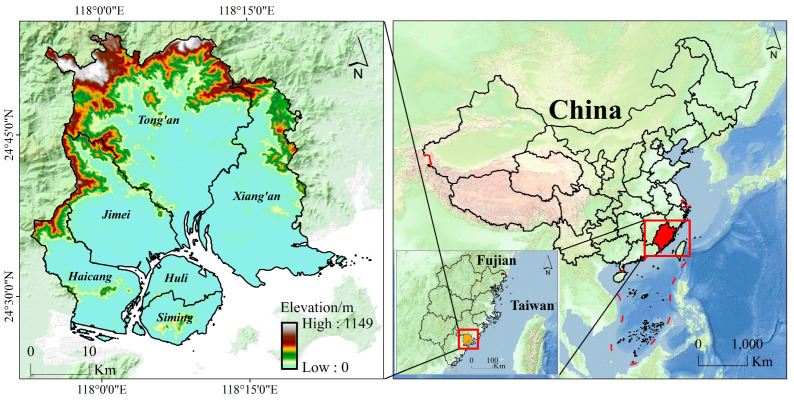
Geographic location of the study area.

Xiamen, comprising Xiamen Island, Gulangyu Island, and various islets and reefs, is divided into six municipal districts. The city’s topography is characterized by coastal plains, terraces, and hills, with slopes descending from northwest to southeast. Various landforms such as mesas, hills, terraces, plains, and mudflats contribute to its diverse landscape [35]. Geologically, Xiamen is positioned at the eastern periphery of the East Min Volcanic Fault Zone and southwest of the Southeast Min Coastal Metamorphic Belt. The area is characterized by three fracture orientations: north–northeast, northwest, and east–west, which contribute to the formation of the Southeast China Coastal Seismic Belt. The region is subjected to a subtropical oceanic monsoon climate, exhibiting mild and rainy weather conditions with an average annual rainfall of 1200 mm, predominantly occurring between May and August [36]. The location of the study area is illustrated in Figure 1.

### 2.2. Data

This study used 80 Sentinel-1A images covering Xiamen from May 2017 to December 2023, acquired in terrain observation by progressive scans (TOPS) imaging mode and processed as single look complex (SLC) data. The data had a spatial resolution of 5 × 20 m, using VV polarization mode, ascending orbit direction, and a revisit period of 12 days. The radar operated in C-band (wavelength of 5.6 cm), with an interferometric wide swath (IW) imaging mode. The pixel size was 13.95 m (azimuthal) × 2.33 m (lateral view), and the central incidence angle of the radar wave was approximately 40.60°.

To facilitate the processing of Sentinel-1A satellite imagery, this study obtained digital elevation model (DEM) data and precise orbit information. These datasets were utilized to correct the terrain-induced phase biases [37,38] and correct systematic errors resulting from orbit inaccuracies [39], respectively. To investigate the factors contributing to surface deformation in Xiamen, this study gathered data of fault zones, seismic focuses, precipitation patterns, reservoir water levels, and groundwater reserves. A comprehensive detailing of the data employed in this study is provided in Table 1.

## 3. Methods

**Figure 2:**
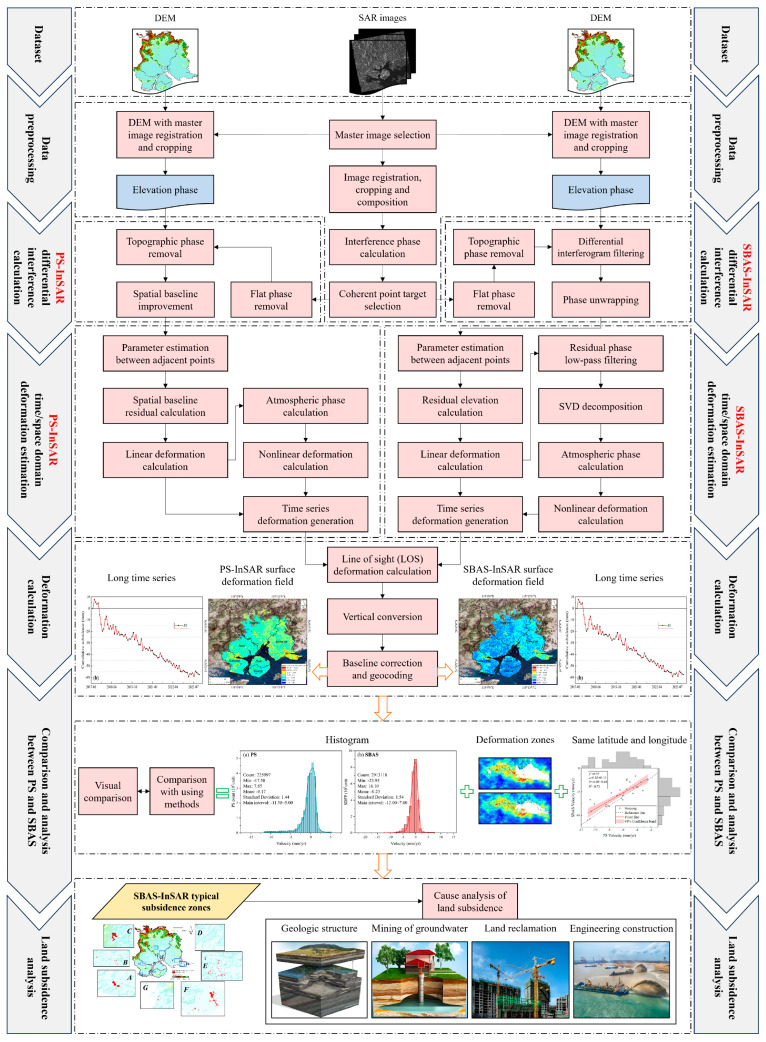
Overall technical methodology flow.

The flowchart of this study is presented in Figure 2. Initially, PS-InSAR and SBAS-InSAR techniques were applied for monitoring the land subsidence of Xiamen, yielding surface deformation fields and time series data. Subsequently, the outcomes of PS and SBAS were compared in terms of histograms, deformation zones, and correlation of homonymous points. Finally, subsidence zones were identified from the optimal surface deformation results, and their associated time series were analyzed to examine the underlying causes of surface subsidence.

### 3.1. PS-InSAR Technique

PS-InSAR acquires time-series SAR images covering the study area. These images are utilized to identify permanent scatterers (PS) points, which exhibit high correlation and stability based on the imagery’s magnitude and phase data. PS points, being relatively insensitive to correlation loss, are utilized to establish a model to solve for deformation phase and separate atmospheric delay phases. This process enables the extraction of deformation information of the target area, and it is an iterative procedure that involves the continuous decomposition and extraction of phase data.

The interferometric phase *φ*_int_ of each resolved element is composed of several factors [40], namely

(1) $$\varphi_{\mathrm{int}}=\varphi_{\mathrm{flat}}+\varphi_{\mathrm{topo}}+\varphi_{\mathrm{def}}+\varphi_{\mathrm{atmo}}+\varphi_{\mathrm{noise}}$$

where *φ*_flat_ is the flat phase, *φ*_topo_ is the terrain phase, *φ*_def_ is the deformation phase, *φ*_atmo_ is the atmospheric delay phase, and *φ*_noise_ is the random noise.

An image is chosen from N images as the master image, with the remaining images of the same acquisition track as slave images. All slave images are carefully aligned with the master image, with the standard deviation smaller than 0.25 pixels to facilitate baseline estimation. Multi-look operation parameters are set, and the magnitude and coherence coefficients are calculated. These are integrated with DEM and the spatial baseline to derive the N − 1 differential interferogram after subtracting the flat phase and terrain phase.

The thresholds for average coherence coefficient, amplitude departure and average amplitude are set to select the coherence points. The coherence point (*x*, *y*) in the *i*th differential interferogram can be expressed as

(2) $$\begin{array}{c} \varphi^{i}(x,y)=[\frac{4\pi }{\lambda }t_{B-A}^{i}\nu (x,y)+\varphi_{\mathrm{nonlinear}}^{i}(x,y)]+\\ \frac{4\pi }{\lambda }\frac{b_{⊥,i}}{r(t_{B-A}^{i})\sin(\theta^{i})}\xi (x,y)+\varphi_{\mathrm{atm}}^{i}(x,y)+\varphi_{\mathrm{noise}}^{i}(x,y) \end{array}$$

where *ξ* and *v* are the elevation error and linear deformation rate, respectively; 4*πtv*/*λ* and *φ*_nonlinear_ are the linear and nonlinear deformation phases, respectively; $\frac{4\pi }{\lambda }\frac{b_{⊥}}{r(t)\sin\theta }\xi$ are the DEM-induced elevation error phases; and *φ*_atm_ and *φ*_noise_ are the atmospheric and noise phases, respectively.

All coherent points are connected through a localized Delaunay triangular network. The atmospheric phases of coherent points within a specified distance threshold (typically 1.2 to 1.5 km) are approximately equal [41]. Thus, for any edge in this triangular grid, the phase difference between its vertices *m* and *n* is linearly modeled as

(3) $$\Delta \varphi_{m,n}(t_{B-A}^{i})=\frac{4\pi }{\lambda }t_{B-A}^{i}\Delta \nu_{m,n}+\frac{4\pi }{\lambda }\frac{b_{⊥,i}t_{B-A}^{i}}{r(t_{B-A}^{i})\sin(\theta^{i})}\Delta \xi_{m,n}$$

The overall phase coherence coefficient method is used to obtain Δ*v* and Δ*ξ*. The deformation phases are then determined by integrating each interconnected edge of the triangular mesh [42]. The atmospheric phase is subsequently estimated and eliminated from the deformation phases using a spatio-temporal filter. This process acquires the average deformation and the cumulative deformation at each coherent point.

### 3.2. PS-InSAR Data Processing

**Figure 3:**
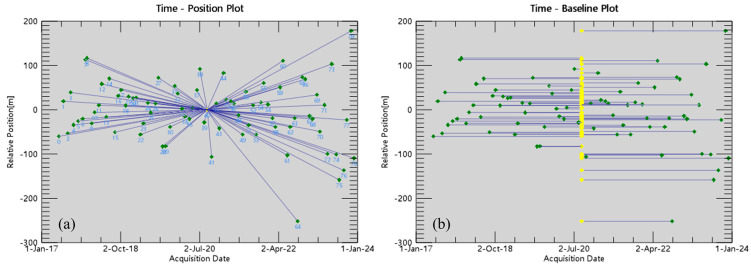
PS interferometric pair connection graph: (**a**) time position graph; (**b**) time baseline graph.

The processing flow of PS-InSAR is shown in Figure 2. The main processing procedures include data preprocessing, generation of the interferometric pair connection graph, interferometric workflow, first inversion, second inversion, and geocoding.

(1). Data preprocessing: We used precise orbit data to correct the orbit information of the original SAR images, and then converted them into SLCs. They were clipped with the study area (.shp) file.
(2). Generation of the interferometric pair connection graph: We selected the image on 1 September 2020 as the super-master image. According to the set critical baseline threshold, 80 SLCs were processed to generate the interferometric pair connection graph (Figure 3).
(3). Interferometric workflow: According to the connection relationship of the interferometric pairs, we performed interferometric workflow processing for each pair of images, mainly including image registration, generation of the interferogram, flattening of the interferogram using the external DEM, and calculation of the amplitude dispersion index. The interferogram at each time point was finally obtained.
(4). First inversion: Based on the identification of a certain number of permanent scatterers, we focused on analyzing the historical phases of these reliable single targets for the first linear model inversion to obtain the displacement rate and residual topography. Then, the synthesized interferogram were flattened.
(5). Second inversion: Based on the result of the first inversion, the atmospheric phase was estimated and removed to obtain the final deformation rate.
(6). Geocoding: The coherence coefficient threshold was set to 0.9. All PS processing results, including the deformation rate, height residual, deformation sequence, KML, vector file, etc., were projected into the map system.

### 3.3. SBAS-InSAR Technique

The Small Baseline Subset InSAR (SBAS-InSAR) method employs small baseline differential interferometry to quantify shape parameters. It effectively aggregates all small baseline interferometric pairs, and then employs the minimum norm criterion for the deformation rate and the Singular Value Decomposition (SVD) technique [24] to retrieve the deformation velocity of the coherent targets and their corresponding time series. The main steps are as follows:

Acquire N + 1 SAR images of the same region arranged chronologically (*t*_0_, …, *t*_N_). Then, select one image as the master image, and align the rest to it. Use the N + 1 SAR images to generate M multi-view differential interferograms. Note that each of the de-entangled differential interferograms has been absolutely corrected by a reference pixel point in a stable region of the map or a reference pixel with a known shape variation.

For the *j*th differential interferogram generated from the SAR image acquired at time *t*_A_ (the slave image) and *t*_B_ (the master image) (*t*_B_ > *t*_A_), the interference phases of the pixels with azimuthal coordinates *x* and distance coordinates *r* can be written as

(4) $$\begin{array}{c} \delta \phi_{j}(x,r)=\phi_{B}(x,r)-\phi_{A}(x,r)\\ \approx \frac{4\pi }{\lambda }[d(t_{B},x,r)-d(t_{A},x,r)]+\Delta \phi_{\mathrm{topo}}^{j}(x,r)\\ +\Delta \phi_{\mathrm{APS}}^{j}(t_{B},t_{A},x,r)+\Delta \phi_{\mathrm{noise}}^{j}(x,r) \end{array}$$

where *j* ∈ (1, …, *M*). *λ* is the central wavelength of the signal. d(*t*_B_, *x*, *r*) and d(*t*_A_, *x*, *r*) are the cumulative deformation of the radar line-of-sight (LOS) direction with respect to d(*t*_0_, *x*, *r*) = 0, at moments *t*_B_ and *t*_A_, respectively. $\Delta \phi_{\mathrm{topo}}^{j}(x,r)$ represents the residual topographic phases in the differential interferograms. If high-precision DEMs used in differential interferometry effectively eliminate most terrain phases, the residual terrain information in differential interferograms is minimal and can be neglected during the solution phase. $\Delta \phi_{\mathrm{APS}}^{j}(t_{B},t_{A},x,r)$ is the atmospheric delay phase. $\Delta \phi_{\mathrm{noise}}^{j}(x,r)$ denotes the decoherent noise (e.g., system thermal noise). Without considering the atmospheric delay phase, residual terrain phase and noise phase [29], Equation (4) can be simplified as

(5) $$\delta \phi_{j}(x,r)=\delta_{B}(x,r)-\delta_{A}(x,r)\approx \frac{4\pi }{\lambda }[d(t_{B},x,r)-d(t_{A},x,r)]$$

In order to obtain a physically meaningful settling sequence, the phase in Equation (5) is expressed as the product of the average phase velocity and time between two acquisition times:

(6) $$\nu_{j}=\frac{\phi_{j}\;-\phi_{j-1}}{t_{j}\;-t_{j-1}}$$

The phase value of the *j*th interferogram can be written as

(7) $$\sum_{k=t_{A,j}+1}^{t_{B,j}}\;(t_{k}\;-t_{k-1})\nu_{k}=\delta \phi_{j}$$

which is the integral of the speed of each time period over the master and slave image time intervals. It can be written in matrix form as

(8) Bv=δφ

Equation (8) is an M × N matrix. Since the differential interferogram with small baseline set adopts a multi-master image strategy, the matrix ***B*** is prone to rank loss. The generalized inverse of matrix ***B*** can be computed using the SVD method. This yields the least squares solution for the velocity vector. Subsequently, the shape variables for each time period can be determined by integrating the velocities over the respective time intervals.

### 3.4. SBAS-InSAR Data Processing

**Figure 4:**
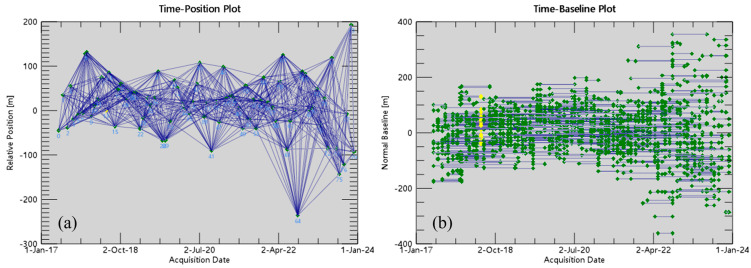
SBAS interferometric pair connection graph: (**a**) time position graph; (**b**) time baseline graph.

The processing flow of SBAS-InSAR is similar to that of PS-InSAR, as shown in Figure 2. The main processing procedures include data preprocessing, generation of the interferometric pair connection graph, interferometric workflow, orbit refinement and reflattening, first inversion, second inversion, and geocoding.

(1). Data preprocessing: We used precise orbit data to correct the orbit information of the original SAR images, and then converted them into SLCs. They were clipped with the study area (.shp) file.
(2). Generation of the interferometric pair connection graph: We conducted interferometric pair matching processing for 80 SAR images. The maximum percentage of the critical baseline was set to 45%, and the maximum time baseline was set to 365 days. We selected the image on 8 June 2018 as the super-master image and optimally combined all the images to form a short baseline set (Figure 4).
(3). Interferometric workflow: We successively conducted interferometric processing on all the pairs in the connection graph, including SLC images pair registration, interferogram generation, flattening the terrain using the external DEM and the terrain phase, differential interferogram filtering, coherence calculation, and phase unwrapping. The threshold of the unwrapping correlation coefficient was set to 0.2. The interferogram, coherence graph, and phase unwrapping graph were finally generated.
(4). Orbit refinement and reflattening: We selected the graphs from (3) with high coherence, good unwrapping, and no deformation and residual terrain fringes. The stable areas far away from the deformation were used as ground control points (GCPs) for orbit refinement and reflattening, which were used to estimate and remove the residual constant phase and the phase ramp that still exists after unwrapping.
(5). First inversion: Firstly, the unwrapped phase was optimized and reflattened. Secondly, based on the orbit refinement and reflattening, we used the SVD algorithm to estimate the deformation rate and the residual topography. Finally, we conducted a secondary unwrapping to obtain the preliminary deformation rate.
(6). Second inversion: On the basis of the deformation rate obtained from the first inversion, we conducted phase optimization, reflattening, and atmospheric filtering. Then, the atmospheric phase was estimated and removed to obtain more accurate displacement.
(7). Geocoding: We performed geocoding on the results of the second inversion of SBAS to obtain the surface deformation results in the line of sight (LOS) direction.

## 4. Results

**Figure 5:**
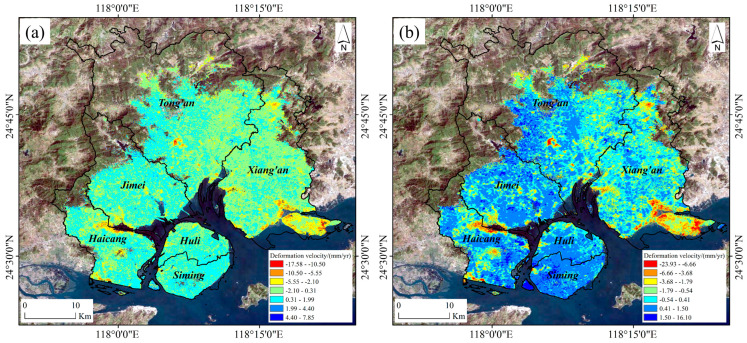
Annual surface deformation of Xiamen from May 2017 to December 2023 derived by (**a**) PS-InSAR and (**b**) SBAS-InSAR.

The annual surface deformation field of Xiamen from May 2017 to December 2023 is derived by PS-InSAR and SBAS-InSAR techniques separately, as shown in Figure 5.

### 4.1. Comparison of PS and SBAS Monitoring Results

From the PS-InSAR and SBAS-InSAR surface deformation fields, several findings are obtained. (1) The average deformation rates of Xiamen obtained by PS-InSAR range from −17.58 to 7.85 mm/yr, while those by SBAS-InSAR span from −23.93 to 16.10 mm/yr. (2) Both PS-InSAR and SBAS-InSAR results reveal similar surface deformation patterns and subsidence funnel distributions. The spatial distribution characteristics exhibit substantial alignment, indicating a high level of consistency between the two techniques. (3) SBAS-InSAR technique demonstrates a significantly higher density of coherent points compared to PS-InSAR, implying that SBAS-InSAR can enhance the interferometric coherence and extract more coherent points [43]. The following is a more accurate and detailed comparison between the two surface deformation monitoring results in terms of histograms, deformation zones and correlation of homonymous points.

#### 4.1.1. Histograms

**Figure 6:**
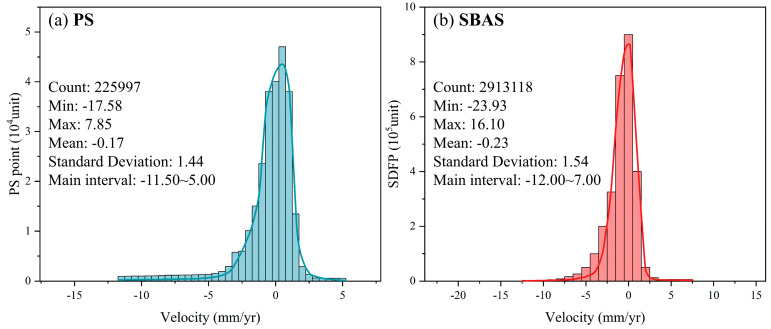
Histogram of deformation rate for (**a**) PS and (**b**) SBAS.

In the absence of concurrent leveling data, this study analyzed the PS points acquired through the PS technique and the Scatterer Displacement Field Points (SDFPs) obtained via the SBAS technique and plotted the histograms illustrating the average deformation rates (Figure 6).

As Figure 6 shows, the average deformation rate across the entire deformation zone obtained by the PS technique primarily falls within the range of −11.50 to 5.00 mm/yr, while that got by SBAS technique is in the range of −12.00 to 7.00 mm/yr. The PS technique generated 225,997 PS points, with 95.29% exhibiting deformation rates between −5.00 and 5.00 mm/yr, 5.16% in the range of −17.58 to −5.00 mm/yr, and 0.45% in the range of 5.00 to 7.85 mm/yr. The SBAS technique generated 2,913,118 SDFPs, where 95.47% displayed deformation rates between −5.00 and 5.00 mm/yr, 4.51% were in the range of −23.93 to −5.00 mm/yr, and 0.02% were in the range of 5.00 to 16.10 mm/yr. These results corroborate the robust consistency of the PS and SBAS techniques and underscore the superior coherence of the SBAS technique.

#### 4.1.2. Deformation Zones

The surface deformation fields identified by the PS and SBAS techniques show nearly identical deformation trends and subsidence funnel distribution. Consequently, five deformation zones from six municipal districts were selected to compare the average deformation rates derived by the two techniques. The results in Table 2 show that the average deformation rates obtained by the two techniques closely match. However, the PS results have a resolution lower than that of the SBAS results. This reaffirms the stronger coherence of the SBAS technique.

#### 4.1.3. Correlation of Homonymous Points

**Figure 7:**
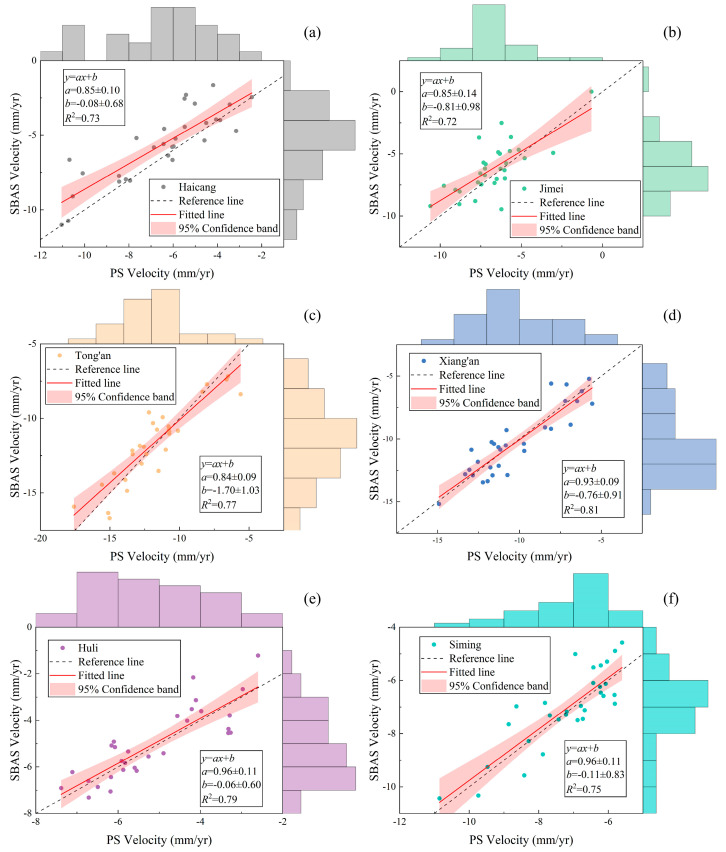
The correlation between the deformation rates obtained through the PS and SBAS techniques in (**a**) Haicang, (**b**) Jimei, (**c**) Tong’an, (**d**) Xiang’an, (**e**) Huli, (**f**) Siming.

Utilizing ArcGIS, we delineated homonymous points within each of the six municipal districts of Xiamen. These homonymous points theoretically correspond to locations with the same latitude and longitude, with an actual error threshold of ≤10^−3^. We analyzed the correlation between the deformation rates obtained through the PS and SBAS techniques (Figure 7). The correlation coefficient *R*^2^ is 0.73 for Haicang, 0.72 for Jimei, 0.77 for Tong’an, 0.81 for Xiang’an, 0.79 for Huli, and 0.75 for Siming. These coefficients suggest that the deformation results obtained through the two techniques exhibit a robust correlation and consistency, confirming the reliability of the data.

### 4.2. Definition of Typical Subsidence Zones

The comparison between the PS and SBAS monitoring results suggests that the SBAS technique yields a significantly higher density of coherent points than the PS technique, despite the strong consistency of the deformation areas identified by the two techniques. This implies that the SBAS technique exhibits superior coherence. Considering these factors, the surface deformation monitoring results obtained by the SBAS technique are superior to those obtained by the PS technique. Therefore, the SBAS technique was utilized to analyze the causes of land subsidence in this study.

**Figure 8:**
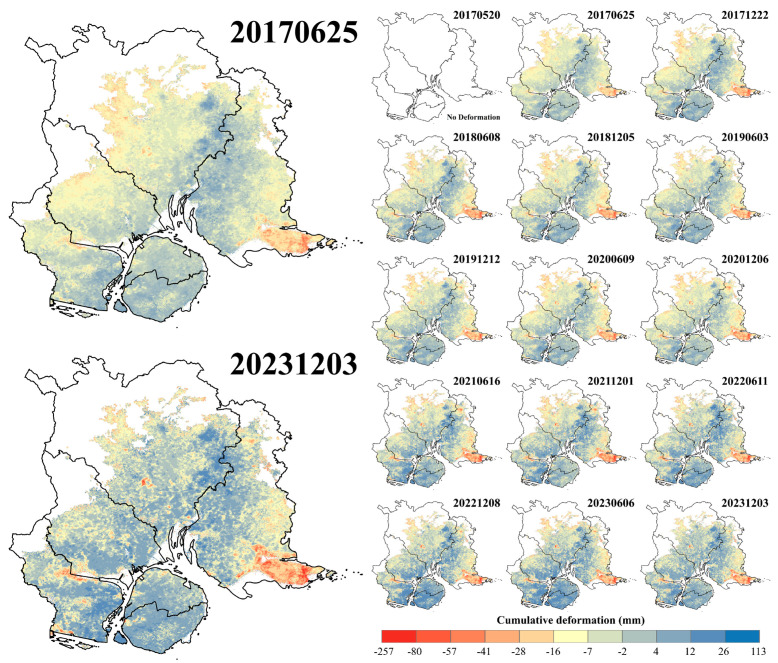
Time series of cumulative deformation in the study area from May 2017 to December 2023. As the cumulative deformation at each acquisition time are referenced to the initial SAR image acquired on 20 May 2017, the initial cumulative deformation result is not shown as a deformation graded rendering.

The SBAS-InSAR geocoding results were processed to derive the cumulative deformation for each SAR image acquisition time, subsequently generating deformation time series through a phase-to-deformation conversion (Figure 8). Figure 8 reveals that the most pronounced subsidence is observed in the southwest (Maluan Bay) and southeast (Dadeng Island) regions. Between 20 May 2017 and 3 December 2023, the cumulative subsidence in these areas exceeds 250 mm.

**Figure 9:**
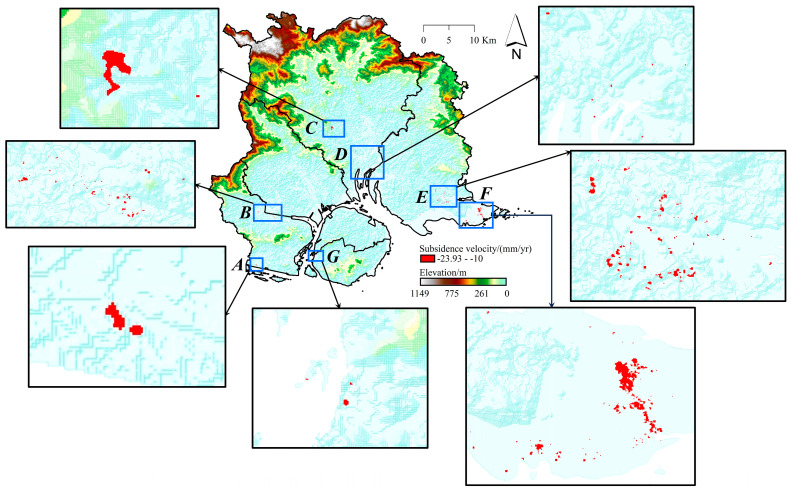
Spatial distribution of 7 typical subsidence zones. *A* is Yuanhai terminal in Haicang District, *B* is Maluan Bay in Haicang District, *C* is Xitang in Tong’an District, *D* is Guanxun in Tong’an District, *E* is Jiuxi entrance and Yangtang in the southern part of Xiang’an District, *F* is the southeastern part of Dadeng Island in Xiang’an District, and *G* is Yundang Lake in Siming District.

We eliminated the surface rise components in the SBAS deformation results and retained only those indicating subsidence. According to the *Technical Specification for Data Processing of Land Subsidence Interferometric Radar*, as outlined in the Geological Survey Technical Standard of China Geological Survey, a subsidence rate less than 10.00 mm/yr is classified as low severity, and that between 10.00 and 30.00 mm/yr is classified as moderate severity. Xiamen’s overall subsidence rate is below 10.00 mm/yr, but it experiences moderate-severity subsidence in some regions. In this study, the regions with subsidence rates between 10.00 and 30.00 mm/yr are designated as the typical subsidence zones, and we found seven such regions, as shown in Figure 9. Large-scale subsidence clusters are observed in zones *A*, *C*, and *F*, while zones *B*, *D*, *E*, and *G* exhibit more dispersed subsidence. These zones are primarily situated along coastlines, urbanized regions, and areas subjected to land reclamation.

## 5. Discussion

Many factors are associated with the subsidence of Xiamen. Next, we will discuss the causes of land subsidence in Xiamen in terms of groundwater extraction, geological structure, land reclamation, and engineering construction.

### 5.1. Impact of Groundwater Extraction on Land Subsidence

Groundwater extraction mainly contributes to land subsidence, causing surface cracks and structural damage [44]. Xiamen, an economic hub, faces challenges due to its underdeveloped surface water system and small, steep watersheds. To meet the great water supply demand, groundwater is excessively extracted. According to the survey, Xiamen’s groundwater storage plummeted by 28% from 2019 to 2020 [45]. The over-exploration of groundwater together with the local geological structure results in ground deformation, fissures, and building damage, severely disrupting daily life and posing significant hazards [44,45,46]. Additionally, it causes seawater intrusion and worsens land subsidence [47].

Groundwater and surface water are interconnected, with groundwater replenishing rivers and lakes during dry seasons and absorbing surplus precipitation during rainy periods, sustaining the balance of the water cycle. To evaluate the influence of groundwater extraction on subsidence, we took direct and indirect approaches to analysis: we directly examined the contribution of groundwater extraction to subsidence, and indirectly discussed the correlation between precipitation, surface water (reservoir level), and subsidence.

**Figure 10:**
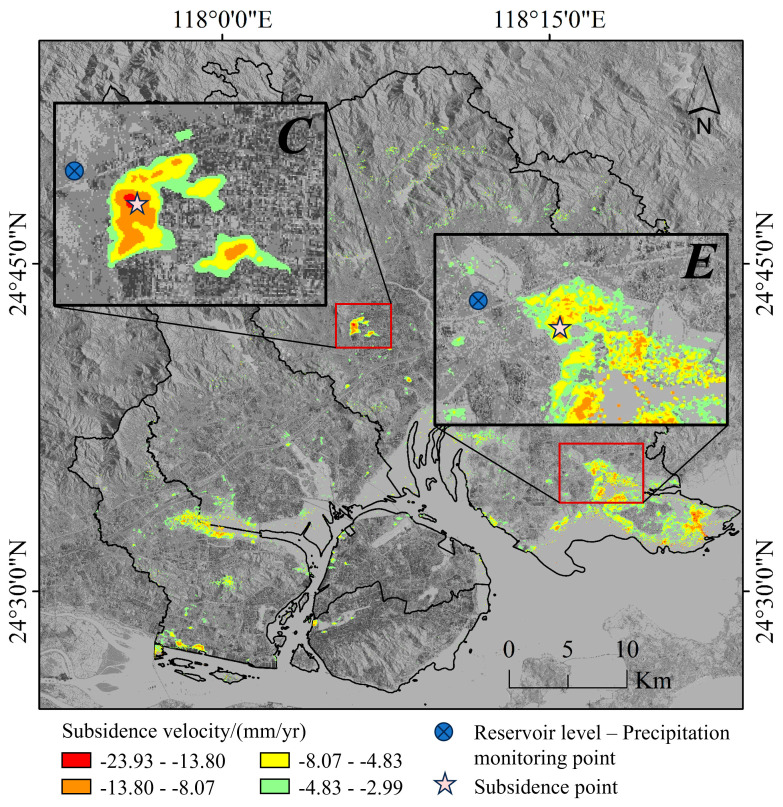
Spatial distribution of reservoir level-precipitation monitoring points and subsidence monitoring points.

We selected the typical subsidence zones with reservoirs for the experiment, which are subsidence zones *C* and *E*. We gathered groundwater, reservoir level, and precipitation data of the areas. Additionally, the subsidence monitoring point data were collected, as illustrated in Figure 10.

(1) Direct analysis

**Figure 11:**
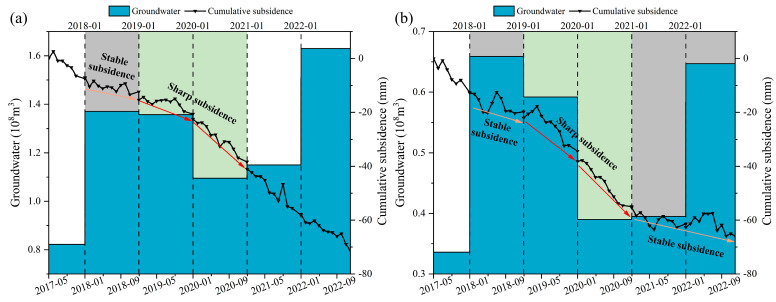
Relationship between groundwater storage and cumulative subsidence in (**a**) zone *C* of Tong’an district and (**b**) zone *E* of Xiang’an district.

The groundwater data and subsidence monitoring points observations were analyzed and are shown in Figure 11. We found that the following: (1) The groundwater level in both Tong’an and Xiang’an districts experienced rise from 2017 to 2018, and then fell between 2018 and 2020, and finally rose between 2020 and 2022. (2) The groundwater level rose from 2017 to 2018, which led to slowing down of land subsidence in 2018. From 2018 to 2020, the fall in groundwater level resulted in the acceleration of subsidence in 2019 and 2020. From 2020 to 2022, the groundwater level rose, with subsidence remaining prominent in 2021 and 2022 in Figure 11a, but slowing down in Figure 11b. Overall, groundwater level rise is associated with a decrease in subsidence rate, while a fall in groundwater level is linked to subsidence acceleration. Xiang’an District exhibits a stronger correlation with this pattern compared to the Tong’an District.

(2) Indirect analysis

**Figure 12:**
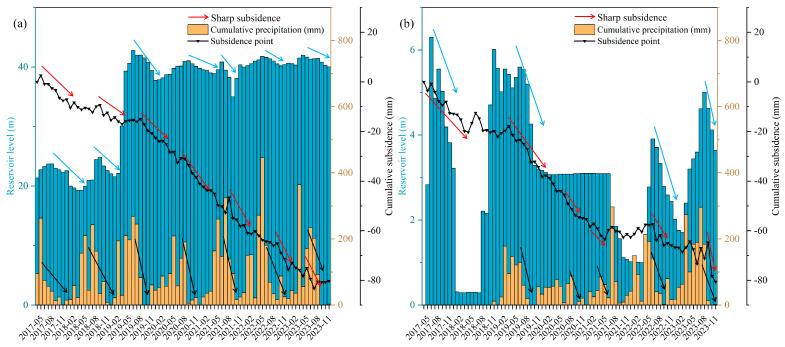
Relationship between reservoir level, cumulative precipitation and cumulative subsidence in (**a**) zone *C* of Tong’an district and (**b**) zone *E* of Xiang’an district.

We analyzed the reservoir water level records, precipitation data and subsidence values in monitoring points, as shown in Figure 12. We obtained some findings: (1) Precipitation exhibits a seasonal cyclic pattern, influencing the reservoir water level in a similar pattern. (2) Periods of decreased precipitation or reservoir water level coincide with increased variability or abrupt changes in cumulative subsidence, as highlighted by the red arrows. This indicates that precipitation has a great influence on land subsidence. This is because reduced rainfall leads to reduced water volume in streams and reservoirs, prompting increased groundwater extraction to meet daily agricultural and industrial needs. Prolonged extraction leads to a sustained declining regional groundwater level, triggering abnormal land subsidence.

In conclusion, both direct and indirect analysis reveal that groundwater extraction exerts a significant influence on land subsidence. A rise in groundwater level corresponds to decreased land subsidence, whereas a fall in groundwater level is associated with accelerated land subsidence.

### 5.2. Impact of Geological Structure on Land Subsidence

Xiamen is situated at the junction of volcanic and metamorphic zones, crisscrossed by faults, forming a rupture tectonic framework. Seismic activity decreases towards the inland and northwest, yet coastal Fujian has a moderate to strong earthquake background [48,49]. Xiamen has small seismic activities, but those earthquakes can significantly impact land subsidence, posing a threat to infrastructure and public safety.

**Figure 13:**
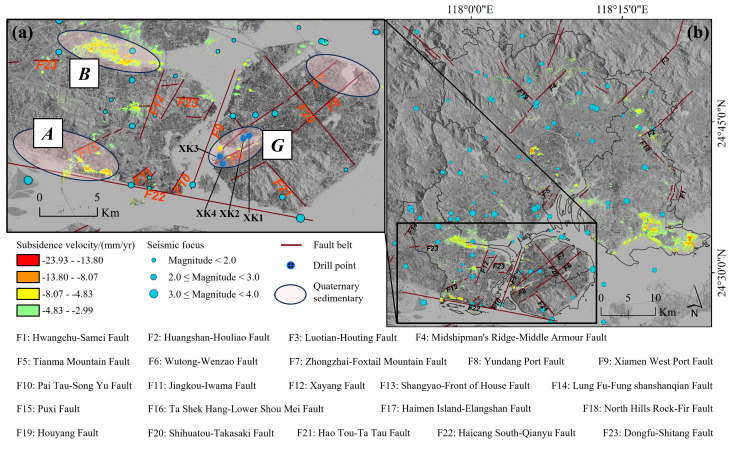
(**a**) Spatial distribution of fault belts, seismic focuses, drilling points and subsidence zones on Xiamen Island; (**b**) spatial distribution of fault belts, seismic focuses and subsidence zones in Xiamen. F1 to F23 are the numbers of the fault belts.

In Xiamen and its surrounding region, there are three groups of fault tectonics, totaling 23 fault belts, as detailed in Figure 13. From January 1981 to September 2023, seismic activities in the region include 32 earthquakes of magnitude < 2.0, 46 earthquakes of magnitude 2.0 to 2.9, and 15 earthquakes of magnitude 3.0 to 3.9. We superposed fault belts and seismic focuses onto the subsidence zones (Figure 13) and found that fault belts and seismic focuses are concentrated within or near subsidence zones *A*, *B*, and *G*.

**Figure 14:**
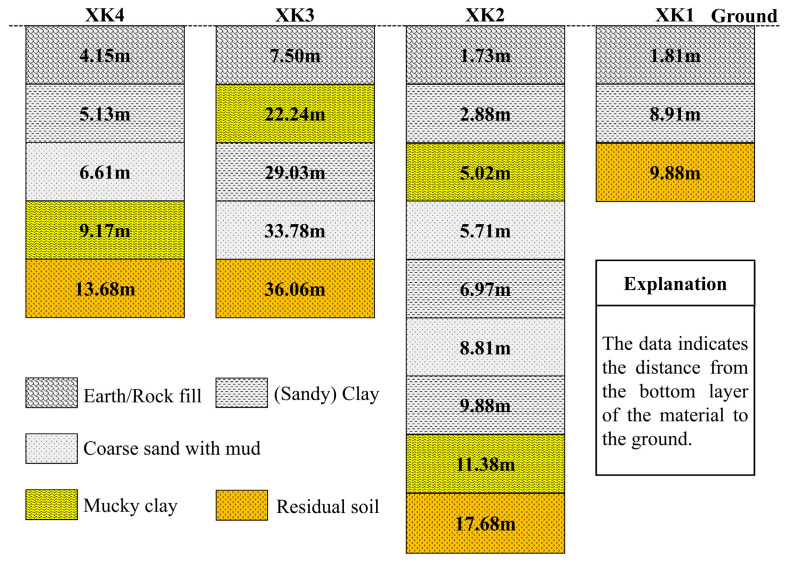
Comparison of the geological structure of the drill hole layering in the Yundang Port Fault Basin, which is modified from the literature [50].

From a lithological perspective, clay layers are the primary cause of subsidence [44]. Scholars [50] have conducted fault detection, interpretation, identification, and localization in Xiamen. They analyzed the three-dimensional stratigraphy and tectonics of Xiamen Island boreholes, and performed cross-fault borehole investigations. The distribution of drilling points is presented in Figure 13a, and a comparison of the geological structures of the Yundang Port Fault Basin boreholes in stratified formations is provided in Figure 14. They found that the typical sedimentation of Yundang Lake in the Siming District lies on the fourth sedimentation layer, and Zone *G* is situated within Quaternary deposits above fault belts F6, F7, F8, and F21. The occurrence of three earthquakes of magnitude 2 or higher in the vicinity, combined with clay layers, suggests a high susceptibility to land subsidence in these areas. Zone *A* and Zone *B*, typical subsidence zones, are also situated within Quaternary deposits. Minor earthquakes with magnitudes of up to 4.0 have been recorded in their vicinity. Zone *A* is proximate to fault belt F22. All these suggest these two zones are prone to subsidence. In conclusion, geological structure significantly influences land subsidence. The regions with dense fault belts and seismic focuses are particularly prone to subsidence.

### 5.3. Impact of Land Reclamation on Land Subsidence

**Figure 15:**
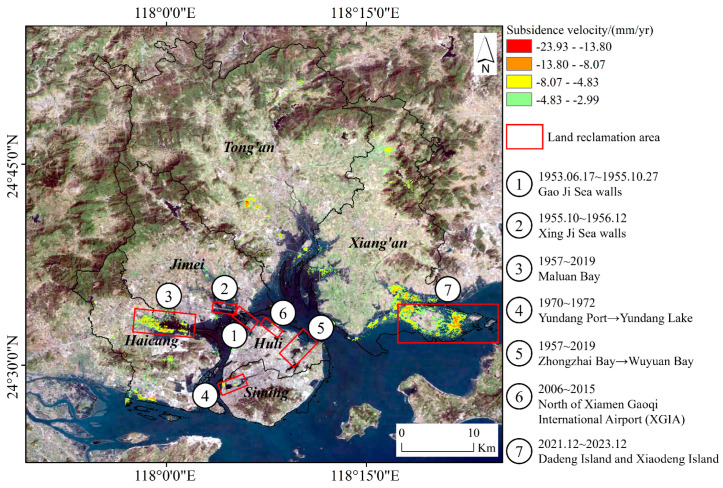
Spatial distribution of large-scale reclamation projects and land subsidence in Xiamen.

Generally, land reclamation, driven by the need for land, marine resources, coastal protection, and aesthetic considerations, has intensified [51]. This practice significantly impacts the coastal zone, modifying geological, hydrogeological, and soil structures and leading to seawater intrusion, land subsidence, and ecological disruption [52,53]. Xiamen, a coastal city, has a long history of land reclamation, particularly notable in the 20th century [54]. The Xiamen Gaoji Seawall, completed in 1955, was a pivotal project that transformed Xiamen from an island to a peninsula. Figure 15 shows Xiamen’s large-scale reclamation areas.

**Figure 16:**
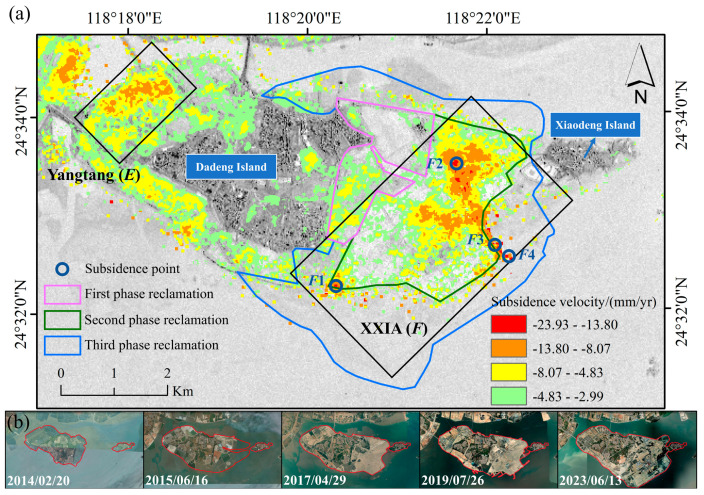
(**a**) Spatial distribution of the area of land subsidence and the three phases reclamation projects in Dadeng Island; (**b**) Dadeng Island coastlines from 2014 to 2023 depicted by satellite images.

The reclamation on Dadeng Island has undergone three phases. The first phase was from March 2014 to June 2015, the second phase was completed on 13 February 2017, and the third phase commenced in January 2022. The areas of these reclamation projects are depicted in Figure 16a, all of which are situated within the typical subsidence zone *F*. In this experiment, subsidence points *F*1, *F*2, *F*3, and *F*4 were selected for detailed analysis. Points *F*1, *F*3, and *F*4 are located close to the boundary of the second-phase project area, and *F*2 is situated is located within the second-phase project area.

**Figure 17:**
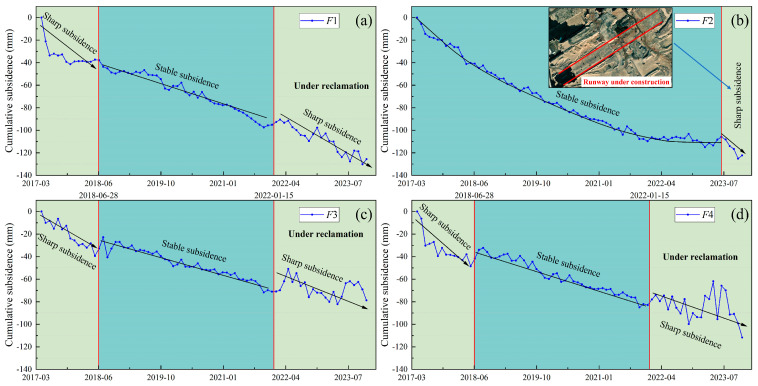
Time-series cumulative subsidence at subsidence points (**a**) *F*1, (**b**) *F*2, (**c**) *F*3 and (**d**) *F*4.

As Figure 17a–d show, points *F*1, *F*3, and *F*4 experienced a rapid subsidence prior to 28 June 2018. Given their proximity to the boundary of the second-phase reclamation project, which concluded on 13 February 2017, the subsidence curves may reflect the consolidation of the filling materials during this period. The initial substantial subsidence was likely due to the consolidation of filling materials between 13 February 2017 and 28 June 2018 [32]. From 28 June 2018 to 15 January 2022, no reclamation activities occurred, leading to subsidence deceleration. After January 2022, when the third phase of reclamation began, a new wave of significant subsidence was observed.

Figure 17b illustrates the subsidence curve of *F*2, which is located inside the second-phase reclamation project area. The second-phase reclamation was completed well before 13 February 2017, so *F*2 underwent the consolidation phase of filling materials earlier than *F*1, *F*3, and *F*4, resulting in slower subsidence between May 2017 and 2023. However, following the construction of the airport runway after 1 July 2023, the land was compacted, showing rapid subsidence.

In summary, land subsidence is intricately linked to reclamation projects, especially during the initial reclamation period, during the consolidation period of the filling material, and post-compaction stages, when substantial subsidence is likely to occur.

### 5.4. Impact of Engineering Construction on Land Subsidence

**Figure 18:**
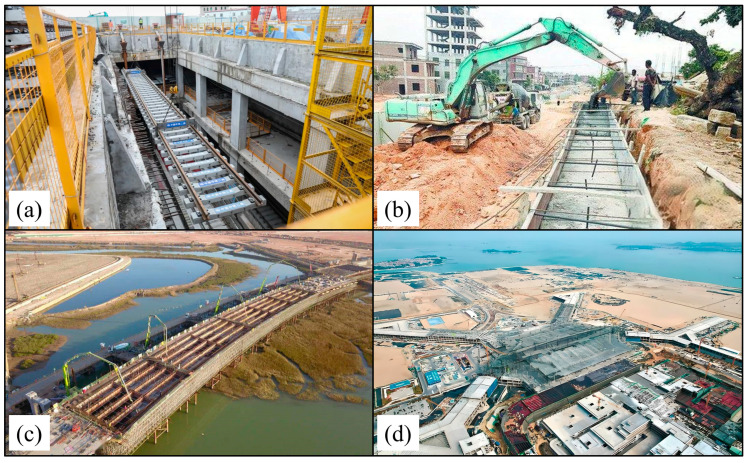
Photos of (**a**) Metro Line 2 under construction, (**b**) Tonglian Road reconstruction, (**c**) Jiuxi Bridge under construction, and (**d**) Xiang’an International Airport under construction.

Land subsidence results from a combination of natural and human-induced factors, with engineering construction being a major contributor [55]. There are completed or ongoing large-scale engineering constructions in typical subsidence zones *A*, *B*, *C*, *D*, *E*, and *F*, such as metros, roads, bridges, airports (Table 3 and Figure 18).

**Figure 19:**
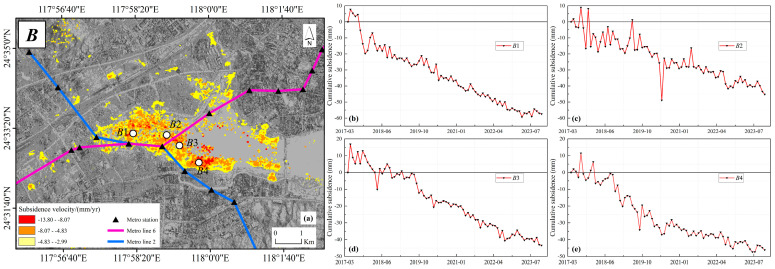
(**a**) Spatial distribution of the Maluan Bay subsidence area and the metro lines passing through it; (**b**–**e**) time-series cumulative subsidence at subsidence points *B*1, *B*2 *B*3 and *B*4.

The Maluan Centre Station in Maluan Bay, located within the Haicang District of Xiamen, is a typical subsidence zone affected by the construction of metro lines. It serves as an interchange for Xiamen Metro Line 2, the under-construction Xiamen Metro Line 4, and Xiamen Metro Line 6. The station features an underground two-level parallel three-island platform. As depicted in Figure 19a, land subsidence in Maluan Bay has been influenced by metro construction, particularly evident around the Maluan Centre Station and along the metro lines. The cumulative subsidence at selected points reaches 60 mm, as illustrated in Figure 19b–e. The impact of metro construction on land subsidence is primarily attributed to the excavation of deep foundation pits and the subsequent underground structural projects. Soil displacement due to excavation and unloading, the deformation of deep foundation pit enclosure structures, and the overall subsidence of underground structures collectively contribute to land subsidence [56,57]. Furthermore, the soil layer in the Maluan Bay area is composed of soft soil and sand, which is saturated with water and leads to a soft and unstable structure. These fragile geological conditions increase the likelihood of land subsidence in this area.

In summary, engineering construction has a significant impact on land subsidence, particularly the alteration of soil structure and the significant ground settlement during new construction.

## 6. Conclusions

Utilizing 80 Sentinel-1A images acquired from May 2017 to December 2023, this study employed PS-InSAR and SBAS-InSAR techniques to comprehensive monitor dynamic changes in land subsidence in Xiamen. After comparison, we selected typical subsidence zones from the results of SBAS-InSAR and investigated the spatio-temporal driving factors of land subsidence. The study yielded the following conclusions:

(1). Although the SBAS technique and the PS technique exhibit strong consistency, the SBAS technique exhibits stronger coherence, making it more advantageous for monitoring surface deformation in Xiamen.
(2). Typical subsidence zones in Xiamen include the Yuanhai Terminal and Maluan Bay in Haicang District, Xitang and Guanxun in Tong’an District, Jiuxi entrance in the southern part of Xiang’an District, Yangtang on Dadeng Island in Xiang’an District, the southeastern part of Dadeng Island in Xiang’an District, and Yundang Lake in Siming District. These areas require special attention for the prevention and mitigation of geological hazards.
(3). This study investigates the spatial and temporal drivers of land subsidence in Xiamen by analyzing four factors that influence land subsidence, in conjunction with the identified typical subsidence zones and their respective long-term time series. Geological structure, particularly in fault zones and seismic focus areas, significantly influence subsidence. Increased groundwater extraction is another significant contributor to land subsidence. Land subsidence is particularly pronounced in reclaimed areas during the initial phase, the consolidation process, and following land compaction. Construction activities alter soil structure and new structures increase the likelihood of land subsidence.

## Figures and Tables

**Table 1:** Information of the data used in this study.

Data	Timespan	Quantities	Unit	Data Sources
Sentinel-1A image	May 2017 to December 2023	80	view	ASF Data Search (https://search.asf.alaska.edu (accessed on 1 December 2023))
DEM	—	—	m	Geospatial Data Cloud (https://www.gscloud.cn/home (accessed on 15 December 2023))
Precision orbit	May 2017 to December 2023	80	—	https://s1qc.asf.alaska.edu/aux_poeorb (accessed on 2 December 2023))
Fault belt	—	23	—	*Earthquake Safety Island*
Seismic focus	January 1971 to December 2023	93	—	*Earthquake Safety Island*
Precipitation	May 2017 to December 2023	80 × 4	mm	XMWCB (http://112.48.134.86:9003/vs4y (accessed on 1 March 2024))
Reservoir level	May 2017 to December 2023	80 × 4	m	XMWCB (http://112.48.134.86:9003/vs4y (accessed on 3 March 2024))
Groundwater	2017 to 2022	6 × 2	10^8^ m^3^	Xiamen Water Resources Bulletin from 2017 to 2022

**Table 2:** Comparison of the deformation zones observed by PS and SBAS techniques.

Zones	Municipal Districts	PS	Velocity [Mode] (mm/yr)	SBAS	Velocity [Mode] (mm/yr)
Maluan Bay	Haicang	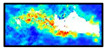	−11.04~2.40 [0.43]	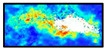	−16.35~6.29 [0.48]
Xitang	Tong’an	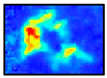	−17.58~2.01 [0.49]	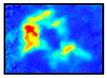	−18.16~2.73 [0.51]
Dadeng Island	Xiang’an	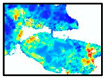	−15.19~3.07 [−1.23]	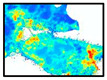	−23.93~10.92 [−1.10]
Shihushan Terminal	Huli	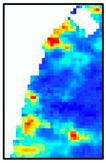	−7.38~2.03 [0.86]	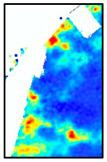	−7.86~4.76 [0.87]
Yundang Lake	Siming	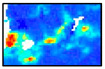	−10.85~3.00 [1.23]	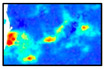	−11.18~4.41 [1.20]

**Table 3:** Engineering construction in typical subsidence zones.

Serial Number	Typical Subsidence Zone	Engineering Construction
*A*	Yuanhai Terminal in Haicang District	On 28 September 2020, Xiamen Yuanhai Terminal railway line officially started construction, and is expected to be completed and opened to traffic in early 2024.
*B*	Maluan Bay in Haicang District	(1) From 1 January 2016 to 17 November 2017, the Maluan Bay section of Metro Line 2 was under construction. (2) From 30 December 2016 to 21 November 2018, the Maluan Bay section of Metro Line 6 was under construction. (3) Metro Line 4 is under construction.
*C*	Xitang in Tong’an District	From October 2015 to May 2021, the Tonglen Road improvement project was underway.
*D*	Guanxun in Tong’an District	(1) Haixiang Avenue is under construction. (2) Metro Line 4 is under construction.
*E*_1_	Jiuxi entrance in the southern part of Xiang’an District	(1) From March 2019 to October 2021, Jiuxi Bridge was under construction. (2) From 2019 to 20 April 2023, the Xidong Bridge was under construction.
*E*_2_	Yangtang in the southern part of Xiang’an District	(1) From August 2004 to August 2018, Dadeng Bridge was under construction. (2) From February 2022 to November 2023, Dadeng Bridge was under an upgrading project. (3)The latter part of Metro Line 3 is under construction. (4) Metro Line 4 is under construction.
*F*	The southeastern part of Dadeng Island in Xiang’an District	On 4 January 2022, the full construction of Xiang’an International Airport began, which is expected to open in 2026.

## Data Availability

All data and materials are available upon request.
